# *Styphnolobium japonicum* Fruit and Germinated Soybean Embryo Complex Extract for Postmenopausal-Symptom Relief

**DOI:** 10.3390/nu16193297

**Published:** 2024-09-28

**Authors:** Jeong-Won Ahn, Hyun-Soo Kim, Kongara Damodar, Hee-Hyun Shin, Kyung-Mi Kim, Jung-Youl Park, Su-Kil Jang, Yeong-Min Yoo, Jae-Chul Jung, Seong-Soo Joo

**Affiliations:** 1 Department of Marine Bioscience, College of Life Science, Gangneung-Wonju National University, Gangneung 25457, Gangwon, Republic of Korea; 0000@gwnu.ac.kr (J.-W.A.);; 2 East Coast Life Sciences Institute, College of Life Science, Gangneung-Wonju National University, Gangneung 25457, Gangwon, Republic of Korea; 3 Huscion MAJIC R&D Center, 331 Pangyo-ro, Seongnam 13488, Gyeonggi, Republic of Korea; 4 Life Science Research Institute, NOVAREX Co., Ltd., Cheongju 28220, Chungbuk, Republic of Korea; 5 Glocal University Project Group, Andong National University, 1375 Gyeongdong-ro, Andong 36729, Gyeongbuk, Republic of Korea

**Keywords:** menopause, *Styphnolobium japonicum* fruit, germinated soybean embryo, ovariectomized rat model, phytoestrogen, medicinal plant

## Abstract

Background/Objectives: Hormonal alterations during menopause result in substantial physiological changes. Although hormone replacement therapy (HRT) is widely used as a treatment strategy for these changes, its use remains controversial due to its associated risks. Plant isoflavones are phytoestrogens that are considered a potential alternative therapy for postmenopausal syndrome. We aimed to investigate the efficacy of ethanolic extracts from *Styphnolobium japonicum* fruit (SJF) and germinated soybean embryo (GSE) in alleviating prominent menopausal symptoms. Methods: A cell model (MCF7 human breast cancer cells) was used to investigate estrogen-like activity. A rat ovariectomy model was used to simulate estrogen depletion after menopause and to evaluate the efficacy of the SJF–GSE complex extract at ratios of 1:1, 1:2, and 2:1. Results: Treatment with the SJF–GSE extract elicited estrogen-like effects, raising pS2 and estrogen receptor α expression in MCF7 cells. The extract was found to contain 48–72 mg/g sophoricoside and 8–12 mg/g soyasaponin 1, identified as active compounds. In ovariectomized rats, the extract effectively reduced body weight and fat content, alleviated vasomotor symptoms, improved vaginal mucosal health, and exerted osteoprotective effects by enhancing bone density and structure, reducing bone-resorption markers and positively altering estradiol levels and lipid profiles. Conclusions: The SJF–GSE extract, working synergistically, provides a safe and effective alternative to HRT for managing postmenopausal symptoms and enhancing bone health, without adverse effects. These findings support the inclusion of SJF and GSE in health-functional foods and underscore the importance of further research into plant-based therapies for menopause.

## 1. Introduction

Menopause, a crucial transition in a woman’s life, is marked by significant hormonal changes that lead to a range of physiological and psychological symptoms. These symptoms, including hot flashes, mood swings, osteoporosis, and changes in metabolic health, can severely affect quality of life [1]. While hormone replacement therapy (HRT) is traditionally used to manage these symptoms, its associated risks have propelled the search for safer natural alternatives [2]. Phytoestrogens, plant-derived compounds found in foods, such as soybeans, flaxseed, and whole grains, mimic the effects of estrogen and present a safer option than traditional HRT [3]. Their capacity to bind to estrogen receptors (ERs) and potentially alleviate menopausal symptoms has attracted significant attention from the scientific community, largely because they present fewer risks than conventional HRT [4].

In the realm of herbal medicine, combining specific plants has shown promise in synergistically alleviating postmenopausal symptoms. Previous studies suggest that such combinations may enhance therapeutic outcomes by interacting in ways that complement and amplify their individual effects, thereby offering a holistic approach to menopause management [5,6]. Germinated soybean embryo, derived from soybeans shortly after the onset of germination but before full sprouting occurs, contains high amounts of bioactive compounds such as isoflavones and soyasaponins [7]. These compounds have been shown to mitigate hormonal imbalances and improve bone health in postmenopausal women [8,9]. Specifically, soyasaponin 1 supports bone health and possesses anti-inflammatory, anti-cancer, anti-obesity, and antioxidant properties [10,11]. *Styphnolobium japonicum* L. (synonym *Sophora japonica* L.) fruit (known as Fructus sophorae) is rich in flavonoids (including sophoricoside) and is known for its antioxidative benefits and hormone-regulatory potential [12]. This study, therefore, focuses on these compounds to better understand their contributions to the observed biological effects.

Here, we aim to elucidate the therapeutic potential of combining ethanol extracts of *S. japonicum* fruit and germinated soybean embryo in an ovariectomized rat model that simulates postmenopausal estrogen depletion. While focusing on the ratio of the extracts in this combination, we examine its efficacy in addressing key menopausal challenges, including vasomotor symptoms, vaginal health, bone integrity, and hormonal balance, positioning this natural solution as a viable and safe alternative to traditional HRT. These findings contribute to the burgeoning field of natural plant-based therapies, offering new insights and potential pathways for improving postmenopausal health.

## 2. Materials and Methods

### 2.1. Sample Preparation

The *S. japonicum* fruit (SJF) and germinated soybean embryo (GSE) complex extracts were provided by NOVAREX Co., Ltd. (Osong, Chungbuk, Republic of Korea). *S. japonicum* fruit extract was derived from the dried ripe fruits of *S. japonicum* L., and germinated soybean embryo extract was prepared from soybeans after controlled germination. Both extracts were obtained using 60 ± 10% food-grade ethanol (Product No. 0019-200L, CAS No. 64-17-5; Korea Ethanol Supplies Company, Seoul, Republic of Korea) via reflux extraction for 4–6 h at 70 ± 10 °C to concentrate key bioactive components, including sophoricoside and soyasaponin 1. The concentrated extracts were filtered, spray-dried to produce the final SJF extract (SJFE) and GSE extract (GSEE) powders, and stored at room temperature. The extracts were dissolved in dimethyl sulfoxide to a concentration of 100 mg/mL and used as a stock solution. To determine the optimal ratio of these extracts, the physiological activity of each extract and the levels of their active compounds were assessed in vitro, at ratios of 1:1, 1:2, and 2:1. For in vivo studies, the final complex extract, containing concentrations of 48–72 mg/g sophoricoside and 8–12 mg/g soyasaponin 1, was prepared by blending SJF and GSE at the identified optimal ratio and yield. A ratio of 1.5:1 (SJF:GSE) satisfied the established criteria for industrial-scale production.

### 2.2. HPLC Analysis

Levels of sophoricoside and soyasaponin 1, the active components of the extracts, were analyzed via 1260 Infinity HPLC using a UV detector (Agilent Technologies, Santa Clara, CA, USA). Chromatographic separation of sophoricoside was performed on a reverse-phase HPLC column (YMC-Triart C18, 4.6 mm × 250 mm, S-5 μm, 12 nm; YMC, Kyoto, Japan) at 30 °C, with a mobile phase comprising 0.1% acetic acid in water (A) and 0.1% acetic acid in acetonitrile (B) at a flow rate of 1 mL/min. The gradient program was as follows: 0–5 min, 15% B; 5–15 min, 15–45% B; 15–17 min, 45–50% B; 17–21 min, 50% B; 21–23 min, 50–15% B; and 23–25 min, 15% B. The injection volume was 5 μL and the detection wavelength was 260 nm.

Chromatographic separation of soyasaponin 1 was performed using a reverse-phase HPLC column (YMC-Triart C18, 4.6 mm × 250 mm, S-5 μm, 12 nm; YMC) at 30 °C. The mobile phase was 0.05% formic acid in distilled water and acetonitrile (at 6:4, *v*/*v*), flowing at 1 mL/min for 25 min under isocratic conditions. The injection volume was 10 μL and the detection wavelength was 210 nm.

### 2.3. Cell Culture

Three distinct cell lines were obtained from the Korean Cell Line Bank (KCLB, Seoul, Republic of Korea) and used to assess the impact of SJFE and GSEE. The human breast cancer cell line MCF7 was cultured in Roswell Park Memorial Institute (RPMI)-1640 medium (Hyclone, Logan, UT, USA), supplemented with 10% fetal bovine serum (FBS; Hyclone), 100 U/mL of penicillin, and 100 µg/mL of streptomycin (Thermo Fisher Scientific, Waltham, MA, USA). Cells were cultured at 37 °C in a humidified atmosphere containing 5% CO_2_. For gene analysis, the cells were seeded at 5.0 × 10^5^ cells/well in 12-well plates and allowed to stabilize for 12 h before being treated with 100 nM 17β-estradiol (E2, Sigma-Aldrich, St. Louis, MO, USA) and 200 μg/mL of each test substance in serum-free medium. Additionally, RAW 264.7 (mouse macrophage) and MG63 (human osteoblast-like) cell lines were used to evaluate nitric oxide (NO) production and overall cellular activity. These cells were maintained in Dulbecco’s Modified Eagle’s Medium (DMEM, Hyclone) supplemented with 10% FBS and antibiotics.

### 2.4. Cell Viability Assay

To evaluate biological activity, cell viability was determined using the Ez-Cytox Enhanced Cell Viability Assay Kit (WST-8; DoGenBio, Seoul, Republic of Korea), according to the manufacturer’s instructions. Briefly, cells were seeded in 96-well plates at a density of 5.0 × 10^4^ cells per well in serum-free DMEM and were treated with various concentrations of the sample. Following treatment for 24 h, the assay reagent was added to each well and the plates were incubated for an additional 2 h at 37 °C. Color intensity, which is directly proportional to the number of viable cells, was quantified by measuring absorbance at 450 nm using a microplate reader (SpectraMax 340, Molecular Devices, San Jose, CA, USA). Viability was expressed as a percentage of the viability in the control group, which was considered to be 100%.

### 2.5. NO and DPPH Assay

We used a mouse macrophage cell line (RAW 264.7) to assess NO production, an essential marker of the inflammatory response, in response to varying concentrations of SJFE, GSEE, and the complex extract. Cells were plated at a density of 1.0 × 10^5^ cells/well in a 96-well plate and allowed to adhere before replacing the medium with 100 μL fresh serum-free medium in each well. The cells were then treated with the test substances at concentrations ranging from 10 to 100 μg/mL, followed by incubation for 1 h. To induce inflammation, 1 μg/mL of lipopolysaccharide (LPS, O55:B5, Sigma-Aldrich) was added, and after 20 h of incubation at 37 °C in a 5% CO_2_ environment, NO production was quantified. This was achieved using the Griess reagent system (Sigma-Aldrich) following the manufacturer’s instructions. A nitrite standard calibration curve (Promega, Madison, WI, USA) was used for quantification. A DPPH (2,2-diphenyl-1-picrylhydrazyl, Sigma-Aldrich) radical scavenging activity assay was performed to screen the antioxidant activity of SJFE and GSEE. In each well of a 96-well plate, 10 μL of each extract was combined with 90 μL of 0.2 mM DPPH solution in methanol. After 10 min of incubation at room temperature, absorbance was measured at 517 nm using a microplate reader. DPPH scavenging activity was calculated using the following formula: scavenging activity (%) = ((control absorbance − sample absorbance)/control absorbance) × 100.

### 2.6. MG63 Activation and RANKL Gene Expression Analysis

We used MG63 osteoblast-like cells and RAW 264.7 macrophage cells to evaluate the modulation of receptor activator of nuclear factor κB ligand (RANK ligand, RANKL) gene expression, a key factor in bone metabolism. The RAW 264.7 cells were initially treated with 1 μg/mL of LPS for 24 h, resulting in a >40-fold increase in NO production relative to the untreated control. This significant increase in NO levels indicates successful macrophage activation. Subsequently, MG63 cells were seeded at a density of 3.0 × 10^5^ cells/well in 12-well plates and allowed to stabilize for 12 h. After stabilization, the cells were cultured in fresh serum-free media and treated with varying concentrations of the test substances, ranging from 10 to 250 μg/mL. One-hour post-treatment, the MG63 cells were exposed to an NO-rich medium derived from activated RAW 264.7 cells, with the NO concentration adjusted to 50 μM. Following a 20-h incubation period in the NO-rich environment, we extracted total RNA from MG63 cells and analyzed gene expression.

### 2.7. qPCR Assay

To analyze changes in gene expression, total RNA was extracted from selected cells or tissues using TRIzol reagent (Thermo Fisher Scientific), following the manufacturer’s guidelines. To synthesize complementary DNA (cDNA), 1.5 µg of total RNA was used as a template. cDNA synthesis was performed using the ImProm-II Reverse Transcriptase System (Promega), following the manufacturer’s protocol. The qPCR reactions were prepared using SensiMix SYBR Hi-ROX PCR Master Mix (Bioline, London, UK) and performed as previously described [13]. The final results are presented in terms of fold change relative to the sham group, providing a clear comparison of gene expression across different experimental conditions. The primer sequences used are listed in Table S1.

### 2.8. Western Blot Analysis

To assess estrogen-like activity in MCF7 cells, the cells were plated at 1.0 × 10^6^ cells/well in 60 mm culture dishes and allowed to stabilize for 12 h. The cells were treated with 100 nM E2 and 200 μg/mL of each test substance in a serum-free medium for 3 h. Total protein from the cells was extracted using radioimmunoprecipitation assay (RIPA) buffer supplemented with a protease and phosphatase inhibitor cocktail (Roche, Basel, Switzerland). Western blotting was performed as described previously [13]. Protein bands were visualized using an enhanced chemiluminescence (ECL) solution (Thermo Fisher Scientific), and luminescence signals were captured using a chemiluminescence imaging system (LuminiGraph II; ATTO, Tokyo, Japan) and quantitatively analyzed using ImageJ software (Version 1.51j; NIH, Bethesda, MD, USA). The results are expressed as relative protein expression and presented as fold-change relative to the control group. The antibodies used are listed in Table S2.

### 2.9. Immunocytochemical Analysis of ERα in MCF7 Cells

MCF7 cells were seeded in four-well chamber slides (1.0 × 10^5^ cells/well) and stabilized for 12 h. After stabilization, the cells were incubated in fresh serum-free medium with 100 nM E2, 200 μg/mL of the complex extract, or 1 µM ER antagonist (ICI 182,780, Sigma-Aldrich) for 24 h. The cells were then fixed in 4% paraformaldehyde (30 min), permeabilized with 0.25% Triton X-100 (20 min), and blocked with 1% BSA to reduce non-specific antibody binding (1 h). Afterward, the cells were incubated with anti-ERα primary antibody (sc-7207, Santa Cruz Biotechnology, Santa Cruz, CA, USA; 1:200 dilution) overnight at 4 °C, followed by incubation with Alexa Fluor 488-conjugated anti-rabbit IgG secondary antibody (Cell Signaling Technology, Danvers, MA, USA; 1:500 dilution) for 1 h at room temperature. Propidium iodide (PI) was used for nuclear counterstaining (20 min at room temperature). Between each step, the slides were washed with phosphate-buffered saline three times after each major step and five times after counterstaining. ERα expression and distribution were analyzed using fluorescence microscopy at 600× magnification (Eclipse Ti-S; Nikon, Tokyo, Japan).

### 2.10. Animal Housing and Ovariectomy

**Figure 1:**
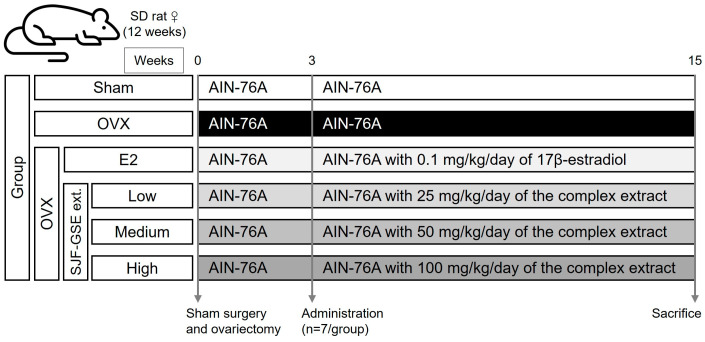
In vivo study design and group allocation. The flowchart illustrates the design of our in vivo study involving adult female Sprague-Dawley (SD) rats. The rats were divided into six groups (*n* = 7 per group): (1) non-ovariectomized control (Sham); (2) ovariectomized control (OVX); (3) positive control group receiving 0.1 mg/kg of 17β-estradiol (E2); groups 4–6 were OVX groups treated with the blended SJF–GSE complex extract at 25 mg/kg (Low), 50 mg/kg (Medium), and 100 mg/kg (High), respectively. After the 3-week recovery period, the rats were provided with custom-manufactured diets containing the specific treatment compounds. The SJF-GSE complex extract was prepared as detailed in the Section 2, with a 1.5:1 ratio of SJF to GSE. SJF, *Styphnolobium japonicum* fruit; GSE, germinated soybean embryo.

We used female Sprague-Dawley (SD) rats, aged 12 weeks and weighing 220–240 g (KOATECH, Gyeonggi, Republic of Korea). The animals were housed in a controlled environment at 21 ± 2 °C, 40–60% humidity, and a 12-h light/dark cycle. Before starting the experiment, the rats underwent a week-long acclimatization period. The rats were divided into six groups (Figure 1): (1) sham-operated (non-ovariectomized control, Sham); (2) ovariectomized control (OVX); (3) OVX treated with 0.1 mg/kg of E2 (positive control); groups 4–6 were OVX groups that received the complex extract at 25 mg/kg (Low), 50 mg/kg (Medium), and 100 mg/kg (High), respectively. The ovariectomy procedure involves dorsal fur shaving, dorsal incision, bilateral ovary removal, and suturing [14]. The Sham group underwent the same surgical procedure, but without ovary removal. Postoperative care was diligently administered to ensure seamless recovery of the animals, without complications, such as incision rupture or inflammation. Only fully recovered animals advanced to the subsequent phase of the experiment (*n* = 7). To establish a reliable OVX rat model for efficacy studies, estrogen depletion was confirmed following ovariectomy. During the 3-week recovery period, the experimental animals were supplied with AIN-76A phytoestrogen-free diet (Harlan Teklad, Madison, WI, USA). The rats were then fed diets formulated according to their group for 12 weeks. Specifically, the Sham and OVX control groups were provided ad libitum diets AIN-76A, whereas the treatment groups were provided with custom-manufactured diets infused with the specified treatment compounds (Daehan Biolink, Chungbuk, Republic of Korea). Body weight was measured twice per week at regular intervals. All animal experiments were approved by the Institutional Animal Care and Use Committee (IACUC) of Gangneung-Wonju National University, Gangneung, Republic of Korea (GWNU-2020-18), and were conducted following the relevant guidelines and regulations and ARRIVE guidelines. Stress was minimized by providing feed mixed with the extracts, and consistent conditions were maintained. No abnormalities were observed, indicating that the changes were due to the treatment.

### 2.11. Measurement of Vasomotor Symptoms

Vasomotor symptoms, which typically appear as initial indicators of menopause in women, were assessed in our postmenopausal rat model. To accurately monitor and evaluate these vasomotor symptoms, we meticulously measured the rectal temperatures of the rats 2 d before their sacrifice. The temperatures were measured at 10-min intervals over a span of 120 min, immediately after 15 min of forced-running on a motorized treadmill (MK-680S, Muromachi Kikai, Tokyo, Japan) at a constant speed of 15 m/min. For each rat, the baseline temperature was established as the average temperature measured 20 min before the forced-running session. Changes in rectal temperature after exercise were recorded for 120 min. To ensure precision and minimize variability in our measurements, rectal temperatures were recorded using a highly accurate electronic thermometer (MT200; Microlife, Taipei, Taiwan). This approach allows for the comprehensive and accurate assessment of vasomotor symptoms, providing crucial insights into the physiological changes associated with menopause in the OVX rat model.

### 2.12. Evaluation of Vaginal Epithelial Cell Changes (Vaginal Cornification)

Vaginal dryness is one of the most common symptoms of menopause. Therefore, we examined changes in the vaginal epithelial cells of each group to assess the efficacy of the extracts in alleviating menopausal symptoms. Vaginal epithelial cells were collected from the vaginal walls of the rats in each group using a sterile swab, immediately before sacrifice. These cells were spread onto a glass slide and stained with Giemsa stain (Sigma-Aldrich). This allowed us to count the number of nucleated and cornified cells to determine the proportion of cornified epithelial cells. A higher proportion of cornified epithelial cells in the vaginal mucosa indicates less vaginal cornification, suggesting that the vaginal mucosa is more flexible and less affected by menopausal dryness.

### 2.13. Assessment of Organ Abnormality and Fat Tissues

Comprehensive organ analyses were conducted to assess the safety of the complex. The process began with a 12-h fasting period, followed by sacrifice via anesthesia using ether. During autopsy, key tissues, such as the liver, spleen, kidneys, stomach, uterus, perirenal fat, and the femurs, were meticulously excised and weighed to detect potential abnormalities or changes. To examine body fat accumulation, histological analysis of adipose tissue was performed. Adipose tissues were fixed in 10% neutral buffered formalin (NBF) for 24 h. The fixed tissues were dehydrated through a graded series of alcohol solutions, cleared in xylene, and infiltrated with paraffin wax. Thin sections (5 µm thickness) were cut from the paraffin-embedded tissue blocks and stained with hematoxylin and eosin (H&E) according to conventional procedures. Lipid droplet area was measured using ImageJ software.

### 2.14. Blood Hormone and Biochemical Analysis

We conducted biochemical analyses to evaluate serum markers indicative of metabolic and liver function in the OVX rat model. Arterial blood was collected for hormone and biochemical analyses, and serum samples were stored at −80 °C. Serum E2 was quantitatively measured using an enzyme-linked immunosorbent assay (ELISA) kit (Cat. No. 501890; Cayman Chemical, Ann Arbor, MI, USA). Serum C-terminal telopeptide (CTx) was quantified using the Rat CTX-1 ELISA kit (NBP2-76634; Novus Biologicals, Littleton, CO, USA). Serum osteocalcin was quantified using a Rat Osteocalcin ELISA kit (NBP2-68153; Novus Biologicals). Biochemical parameters, including alanine aminotransferase (ALT), triglycerides (TG), high-density lipoprotein (HDL), low-density lipoprotein (LDL), lactate dehydrogenase (LDH), calcium, and phosphorus levels, were analyzed using an INTEGRA 400 automatic analyzer (Roche, Mannheim, Germany).

### 2.15. Microscopic Assessment of Trabecular Bone Loss

Microscopic evaluation of trabecular bone loss was performed on the femur. Right femurs were fixed in 10% NBF for 24 h and decalcified using 10% ethylenediaminetetraacetic acid (EDTA) at 4 °C for 14 d. Following decalcification, the bones were embedded in paraffin. From these, sections of 5 µm thickness were cut for detailed examination. The sections were then stained with H&E, a technique commonly used to highlight bone microarchitecture. The resulting stained sections provide a clear visualization of the structural integrity of the bone. The area of bone loss was measured using ImageJ software.

### 2.16. Isolation of Bone Marrow Cells from Femurs

Comprehensive gene expression analyses were performed using the femurs of the OVX rats to elucidate the molecular mechanisms associated with estrogen depletion. After the 12-week treatment period, the rats were euthanized and tissues were collected for analysis. Bone marrow cells were isolated from the left femur using a previously described method, with minor modifications [15]. Briefly, the bones were bisected and placed in microcentrifuge tubes. Bone marrow was harvested via centrifugation at 5000× *g* for 5 min. The cells were then filtered through a 40 µm cell strainer to remove debris, and erythrocytes were lysed using RBC lysis buffer (BioLegend, San Diego, CA, USA). qPCR was used to analyze the expression of genes pertinent to bone tissue metabolism and health.

### 2.17. Assessment of Bone Mineral Contents, Bone Mineral Density, and X-ray Imaging

We evaluated the effects of the complex extract on postmenopausal bone health in the OVX rat model, using dual-energy X-ray absorptiometry (DXA) to determine bone mineral content (BMC) and bone mineral density (BMD) measurement, and X-ray imaging for structural observation (PIXImus II densitometer, GE-Lunar, Madison, WI, USA). The left femur was prepared and analyzed via DXA to quantify both BMC and BMD to assess bone integrity. Preparation involved removing the surrounding muscle and tissue and preserving the bone in sterile saline for accurate measurement. X-ray imaging of the femur revealed structural changes, complementing the quantitative and qualitative analyses and providing a holistic view of bone health.

### 2.18. Statistical Analysis

The results are presented as means ± standard deviation. The significance of differences between experimental groups was analyzed using Student’s *t*-tests or one-way ANOVA followed by Tukey’s post-hoc test, using GraphPad Prism Version 5.01 (GraphPad Software Inc., San Diego, CA, USA). Differences were considered significant at *p* < 0.05.

## 3. Results and Discussion

### 3.1. Optimal Ratio of SJFE and GSEE

In examining the optimal ratio of SJFE and GSEE, we focused on their antioxidative and anti-inflammatory properties and their effects on osteoclastogenesis-related gene expression. Menopause, marked by a significant decrease in estrogen levels due to the cessation of ovarian follicle function, leads to increased oxidative stress within the body. This elevation in oxidative stress not only exacerbates inflammation but also contributes to obesity, which in turn can further enhance oxidative stress, creating a detrimental cycle of metabolic disturbance [16,17]. These molecular alterations have been implicated in the pathogenesis of postmenopausal osteoporosis [18]. At the concentrations used here, the extracts did not affect cell activity (Figure S1). We first examined the extracts’ antioxidant potential using a DPPH scavenging activity assay. This revealed that SJFE exhibited DPPH scavenging activity at a half-maximal inhibitory concentration (IC_50_) of 61.4 ± 11.2 μg, whereas GSEE demonstrated this activity at an IC_50_ of 293.7 ± 15.7 μg (Table 1; Figure S2).

The extracts’ anti-inflammatory potential was assessed by measuring the inhibition of NO production in RAW 264.7 macrophage cells. SJFE and GSEE had median effective dose (ED_50_) values of 206.7 ± 18.1 μg/mL and 34.2 ± 5.7 μg/mL, respectively (Table 1; Figure S3). The optimal concentrations were selected based on their efficacy and non-toxicity, as indicated by the IC_50_ and ED_50_ values, which represent the concentrations at which 50% of the maximal effect was achieved.

The effectiveness of these extracts in modulating bone metabolism was evaluated by examining their effects on RANKL gene expression in MG63 osteoblast-like cells. The inhibitory effects on RANKL expression were significant (*p* < 0.001) at 250 μg/mL of SJFE and 10 μg/mL of GSEE (Table 1; Figure S4). This indicates that SJFE exhibited greater antioxidant activity than GSEE, while GSEE demonstrated superior anti-inflammatory and RANKL-inhibitory activity.

In testing antioxidant activity via the DPPH assay, ascorbic acid treatment was used as the positive control; in contrast, anti-inflammatory effects, including inhibition of NO production and of RANKL expression, were evaluated relative to negative controls. Based on these findings, we hypothesized that the two extracts, with their distinct antioxidative and anti-inflammatory properties, could have complementary effects in alleviating menopausal symptoms. Consequently, we further examined the optimal ratio of SJFE and GSEE and their synergistic effectiveness.

The ratio of SJFE and GSEE that maximizes their synergistic effects was examined. Three ratios of SJFE to GSEE (1:1, 1:2, and 2:1) were tested and their DPPH scavenging, NO production inhibition, and RANKL expression inhibition activity were recorded (Table 1). Based on these assays, a 2:1 ratio of SJFE to GSEE was the most effective, balancing the physiological activity of both extracts.

### 3.2. Impacts of SJFE and GSEE Combinations on MCF7 Cells

**Figure 2:**
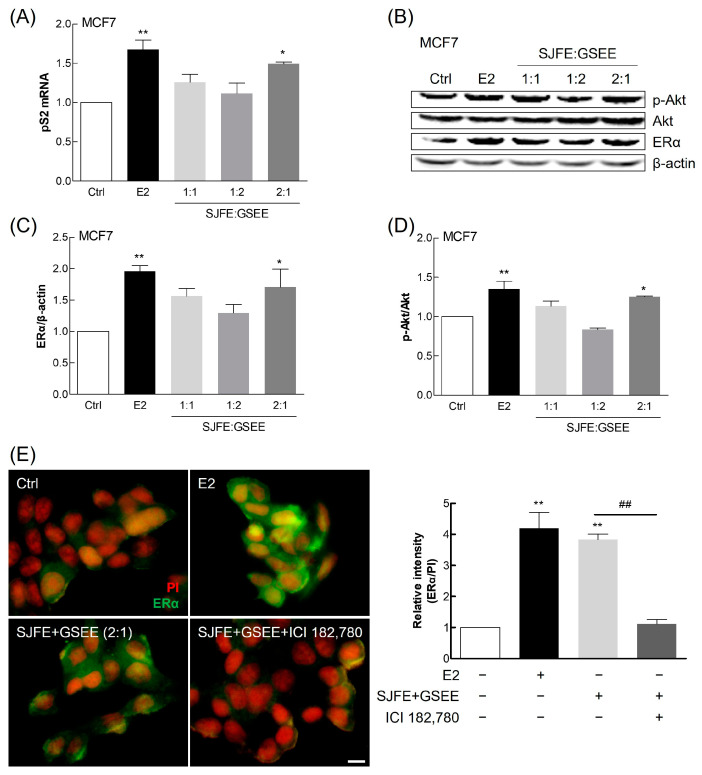
Evaluation of the estrogen-like activity of *Styphnolobium japonicum* fruit extract (SJFE) and germinated soybean embryo extract (GSEE) combinations in MCF7 cells. (**A**) Expression of pS2 mRNA, quantified using qPCR. (**B**–**D**) Representative immunoblots of ERα, phosphorylated Akt (p-Akt), total Akt, and β-actin. (**E**) Immunocytochemical analysis of ERα expression (left panel). The relative ERα/PI ratio is presented as fold-change relative to the control (right panel). 17β-estradiol (E2) was used as a positive control, and ICI 182,780 was used as an estrogen antagonist. Scale bar, 100 µm. Results from three independent experiments are shown as means ± standard deviation. * *p* < 0.05, ** *p* < 0.01, vs. the control group; ## *p* < 0.01, vs. the indicated group. ER, estrogen receptor; PI, propidium iodide.

In humans, ERα and ERβ are pivotal for regulating various physiological processes. ERα predominantly influences reproductive function, whereas ERβ is involved in the central nervous system, cardiovascular system, and bone health [19]. MCF7 cells are commonly used to assess the estrogenic effects of phytoestrogens, owing to their stable estrogen sensitivity and to the reproducibility of these effects in these cells [20]. Menopausal symptoms arise largely from the decline in estrogen levels, which these receptors mediate. Here, we examined the potential of combinations of these extracts, as estrogen substitutes, to alleviate menopausal symptoms by targeting Erα in particular. The expression of the estrogen-responsive gene pS2 was significantly elevated in MCF7 cells treated with a combination of SJFE and GSEE at a 2:1 ratio (Figure 2A). Coincidently, this combination significantly increased ERα expression and Akt activation (Figure 2B–D). These results suggest the efficacy of this combination in mimicking the effects of estrogen, consistent with previous findings highlighting its estrogen-like activity [21,22]. Here, following the guidelines recommended by the Korea Food and Drug Administration, we focused on the expression of the pS2 gene in MCF7 cells, which are known for their responsiveness to estradiol, as well as on ERα activation and Akt phosphorylation. Although other biomarkers could have been analyzed, the selected markers effectively represent the key aspects of estrogenic activity relevant to our research.

Immunocytochemical staining for ERα in MCF7 cells revealed that, even in the absence of estrogen, the SJFE–GSEE complex activated this receptor, indicating the combination’s estrogen-like properties (Figure 2E). This activation was inhibited by ICI 182,780, an estrogen antagonist, confirming the competitive specificity of the interaction between the test substances and ERα [23].

These findings demonstrate that the complex extract (SJFE:GSEE, 2:1) can potentially compensate for estrogen deficiency during menopause, enhancing cellular responses in a similar way to estrogen. The complex extract is therefore a promising agent for addressing menopausal symptoms related to estrogen decline, consistent with previous findings [24,25]. These findings underscore the potential application of this extract in treating menopausal symptoms, opening new avenues for natural health-product development and research into women’s health.

### 3.3. Composition of Active Compounds in the SJF–GSE Extract

**Figure 3:**
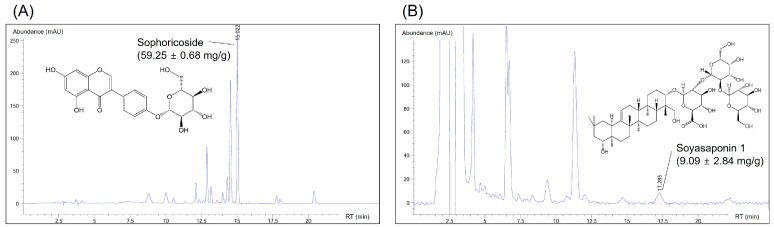
Analysis of active compounds in the *Styphnolobium japonicum* fruit (SJF) and germinated soybean embryo (GSE) complex extract, prepared by blending the SJF and GSE. HPLC was used to analyze sophoricoside (**A**) and soyasaponin 1 (**B**) levels. The compounds were identified and measured by comparing their retention times (RTs) against those of the standards.

We examined the extracts’ active components and marker compounds, sophoricoside and soyasaponin 1. Sophoricoside exhibits a multifaceted pharmacological profile, including estrogenic, anti-inflammatory, antioxidative, antidiabetic, and immunomodulatory activity [12,26]. Soyasaponins are distinguished by their diverse chemical structures and broad-spectrum health benefits, with soyasaponin 1 noted for its anti-inflammatory, anticarcinogenic, hepatoprotective, and antioxidative properties [10,11,27]. We used HPLC to quantify the levels of these compounds (Figure S5). This revealed substantial levels of sophoricoside and soyasaponin 1 within the extract, at 59.25 ± 0.68 mg/g and 9.09 ± 2.84 mg/g, respectively (Figure 3). These results fall within the range (48–72 mg/g sophoricoside and 8–12 mg/g soyasaponin 1) established when analyzing the combinations of SJFE and GSEE, indicating that the composition of the active compounds is equivalent (Table S3). These high concentrations highlight the extracts’ potential roles in ameliorating menopausal symptoms and emphasize the therapeutic relevance of the composition of the complex extract.

### 3.4. Body Weight and Vasomotor Symptom Improvement

**Figure 4:**
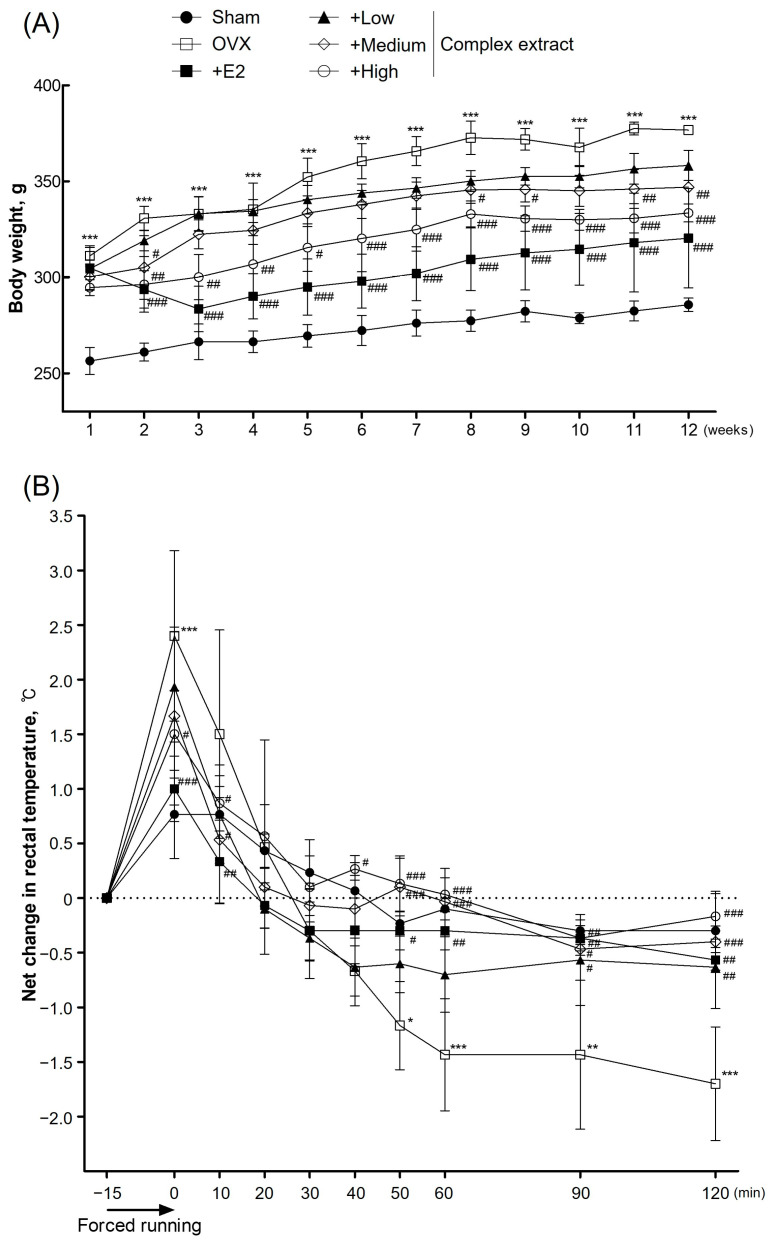
Body weight and vasomotor symptoms in ovariectomized (OVX) rats treated with the *Styphnolobium japonicum* fruit (SJF) and germinated soybean embryo (GSE) complex extract. (**A**) Body weight over 12 weeks. (**B**) Rectal temperature changes during the 120 min post-exercise. The complex extract was prepared by blending SJF and GSE. 17β-estradiol (E2) was used as a positive control. The results are presented as the means ± standard deviation (*n* = 7). * *p* < 0.05, ** *p* < 0.01, *** *p* < 0.001 vs. the sham group; # *p* < 0.05, ## *p* < 0.01, ### *p* < 0.001, vs. the OVX group.

We next evaluated the potential of the complex extract to alleviate menopausal symptoms in the OVX rat model. During the 12-week experimental period, changes in body weight were monitored and vasomotor symptoms were examined as a key indicator of facial flushing during menopause. The estrogen-supplemented positive control group showed the least variation in weight, illustrating the significant role of estrogen in weight regulation after ovariectomy. Notably, the complex extract-treated groups exhibited significantly lower weight gain than the OVX control group (Figure 4A). This is consistent with post-ovariectomy weight-management trends in rodent models, suggesting the effectiveness of the SJF–GSE extract in simulating the weight-regulatory role of estrogen [28,29]. Additionally, we measured changes in vasomotor symptom based on the initial temperature readings (Figure 4B). The rectal temperature of OVX rats changed substantially and remained constant for >2 h. Facial flushing, resulting from capillary congestion in the epidermis, leads to increased skin blood flow, elevated temperatures, and reduced skin moisture [30]. These symptoms are often linked to substantial declines in estrogen levels during menopause, which are believed to reduce the hypothalamic setpoint for body temperature regulation [31]. This suggests that even minor increases in core body temperature could trigger vasomotor symptoms, making their observation a crucial marker for assessing the severity of menopausal symptoms resulting from hormonal depletion. The OVX group exhibited substantial fluctuations in temperature, indicating an unstable post-exercise response. Although the complex extract-treated rats also exhibited temperature variation after exercise, their temperature gradually normalized, following a pattern similar to that observed in the positive control group. This suggests a link between the induced temperature changes in our model and common menopausal symptoms in humans, such as facial flushing and increased body temperature; further, these symptoms were affected by the complex extract, regardless of the dose [32].

### 3.5. Postmenopausal Vaginal Symptoms (Vaginal Keratinization)

**Figure 5:**
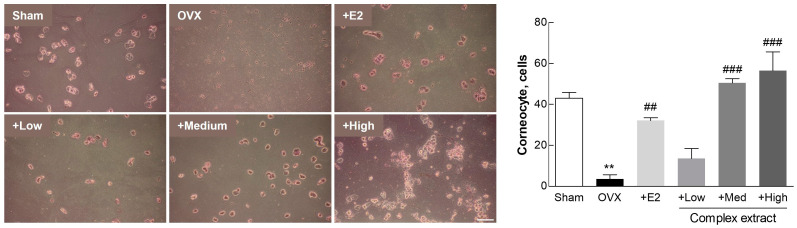
Vaginal cornification in ovariectomized (OVX) rats treated with the *Styphnolobium japonicum* fruit (SJF) and germinated soybean embryo (GSE) complex extract. Representative images of stained vaginal epithelial cells, illustrating the proportion of nucleated and cornified cells (left panel). Counting and comparison of the number of cornified cells (right panel). Scale bar, 100 µm. The complex extract was prepared by blending the SJF and GSE. 17β-estradiol (E2) was used as a positive control. The results are presented as means ± standard deviation (*n* = 7). ** *p* < 0.01, vs. the sham group; ## *p* < 0.01, ### *p* < 0.001, vs. the OVX group.

We examined changes in vaginal epithelial cells, focusing on vaginal cornification as a key indicator. Urogenital symptoms, including vaginal dryness, are prevalent in >50% of menopausal women, primarily owing to hormonal fluctuations during menopause [33]. These hormonal changes often lead to the thinning and cornification of the vaginal lining. To examine the efficacy of our experimental substances in mitigating these symptoms, we analyzed the vaginal epithelial cells from each group. Using Giemsa staining, we quantified the nucleated and cornified cells from vaginal epithelial samples collected using sterile swabs immediately before euthanasia. The proportion of cornified cells serves as an indicator of vaginal health; a higher proportion indicates reduced cornification and improved mucosal flexibility, factors that are essential for maintaining vaginal tissue health during menopause [34,35]. The Medium- and High-dose extract-treated groups displayed higher levels of cornified epithelial cells than the positive control group, indicating amelioration of the vaginal mucosal condition (Figure 5). This suggests that the extracts potentially enhance the flexibility and overall health of the vaginal mucosa in a menopausal model, mirroring the therapeutic benefits observed in other studies [36].

### 3.6. Postmenopausal Changes in Uterine and Adipose Tissue

**Figure 6:**
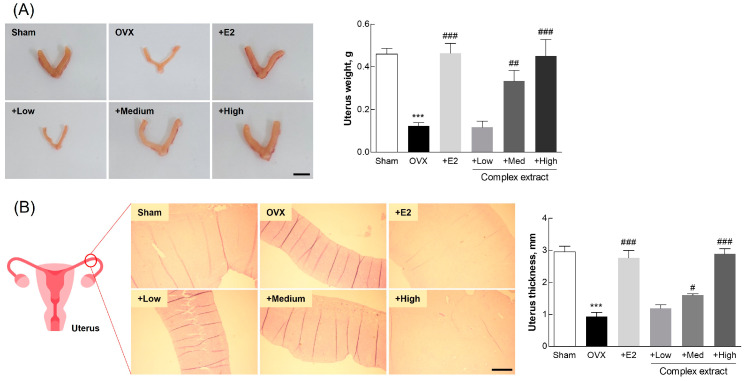
Uterine changes in ovariectomized (OVX) rats treated with the *Styphnolobium japonicum* fruit (SJF) and germinated soybean embryo (GSE) complex extract. (**A**) Representative images of dissected uterine tissue from the rats (left panel). Uterine weight (right panel). Scale bar, 1 cm. (**B**) Representative images of hematoxylin and eosin-stained uterine walls (left panel). Uterine thickness (right panel). Scale bar, 500 µm. The complex extract was prepared by blending the SJF and GSE. 17β-estradiol (E2) was used as a positive control. The results are presented as means ± standard deviation (*n* = 3). *** *p* < 0.001, vs. the sham group; # *p* < 0.05, ## *p* < 0.01, ### *p* < 0.001, vs. the OVX group.

Our investigation into the therapeutic effects of the complex extract on postmenopausal symptoms yielded important insights into changes in uterine and adipose tissue. The complex extract exhibited a favorable safety profile, with no significant changes in organ weight, suggesting its suitability for use without the risk of adverse organ-related effects (Figure S6). Notably, we observed a dose-dependent increase in uterine weight among the treatment groups (Figure 6A), indicating the compensatory estrogenic effects of the complex extract. This increase in uterine weight suggests the potential of this extract to maintain uterine health; this potential is especially relevant under conditions simulating postmenopausal estrogen deficiency [37]. The complex extract-treated groups exhibited an increase in uterine wall thickness (Figure 6B), consistent with our findings on uterine weight, and further supporting the notion that treatment can effectively counteract the negative effects of estrogen deficiency [38]. These findings highlight the therapeutic potential of this extract in mimicking the beneficial effects of estrogen on uterine tissue, thus offering a promising avenue for the management of postmenopausal symptoms.

**Figure 7:**
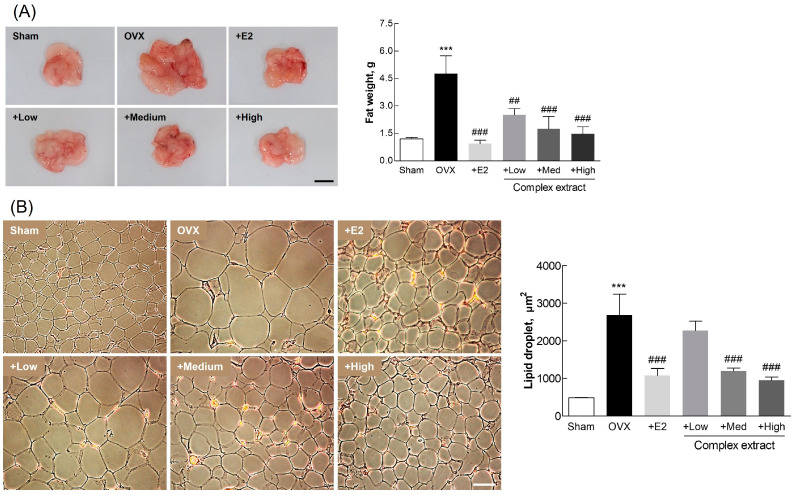
Adipose tissue analysis in ovariectomized (OVX) rats treated with the *Styphnolobium japonicum* fruit (SJF) and germinated soybean embryo (GSE) complex extract. (**A**) Representative images of the perirenal adipose tissue (left panel). Fat weight (right panel). Scale bar, 1 cm. (**B**) Representative images of lipid droplets (left panel). Lipid droplet size (right panel). Scale bar, 100 µm. The complex extract was prepared by blending the SJF and GSE. 17β-estradiol (E2) was used as a positive control. The results are presented as means ± standard deviation (*n* = 3). *** *p* < 0.001, vs. the sham group; ## *p* < 0.01, ### *p* < 0.001, vs. the OVX group.

Following ovariectomy and subsequent treatment, adipose tissue weight was significantly higher in the OVX group than that in the sham group (Figure 7A). This supports the notion that estrogen depletion contributes to increased adiposity. The treatment groups, and especially the High-dose extract-treated group, exhibited adiposity levels comparable to those in the sham group, indicating the complex extract’s effectiveness in countering menopause-associated adipocyte proliferation and lipid accumulation. The observed regulation of lipid-droplet size in the complex extract-treated groups (Figure 7B) supports prior findings regarding the role of estrogen in managing body weight and visceral fat after ovariectomy [39]. These results provide substantial evidence of the ability of the SJF–GSE extract to alleviate menopausal symptoms, including increased adiposity, associated with estrogen depletion.

These findings reveal the potential of the SJF–GSE complex in mitigating the key symptoms of menopause in OVX rats, thus elucidating its potential for managing postmenopausal conditions in humans. The notable improvements observed in uterine and adipose tissue mark a significant step toward developing effective therapeutic strategies to manage postmenopausal symptoms caused by estrogen deficiency.

### 3.7. Blood Biochemical Markers

We examined blood biochemical markers in the OVX rats following the 12-week treatment regimen with varying concentrations of the complex extract (Table 2). E2 levels were significantly elevated in the positive control and High-dose extract-treated groups, indicating the estrogen-mimicking potential of these treatments [40]. Levels of ALT, an indicator of liver health, initially increased in the extract-treated groups but declined with increasing dosage, suggesting a dose-dependent beneficial effect [41]. TG levels were reduced in both the E2-and complex extract-treated groups. The LDL levels observed suggest the possible long-term benefits of these treatments on lipid metabolism and cardiovascular health [42]. The LDH levels, which are indicative of tissue damage and serve as a predictive marker for various cancers—especially breast cancer in postmenopausal women—varied significantly with extract concentration. This suggests that ongoing administration of this extract may improve postmenopausal health [43].

The effect of the extracts on the bone health markers osteocalcin and CTx were examined. Osteocalcin and CTx levels were significantly elevated after ovariectomy, but were decreased in the positive control and complex extract-treated groups, exhibiting a dose-dependent response. When comparing the serum levels of minerals associated with bone health, Ca levels were not significantly altered; P levels, in contrast, were decreased in the OVX group and were dose-dependently elevated in the extract-treated groups, with the High-concentration and sham groups exhibiting similar P levels. This suggests that the complex extract potentially counteracts the bone loss associated with estrogen depletion [44,45]. These results strongly suggest that this complex extract can effectively modulate key biochemical markers in post-OVX rat models, thus highlighting their potential for managing the symptoms and health concerns related to menopause.

### 3.8. Evaluation of Femoral Bone Features: Gene Expression, Histological Staining, and Imaging Analysis

**Figure 8:**
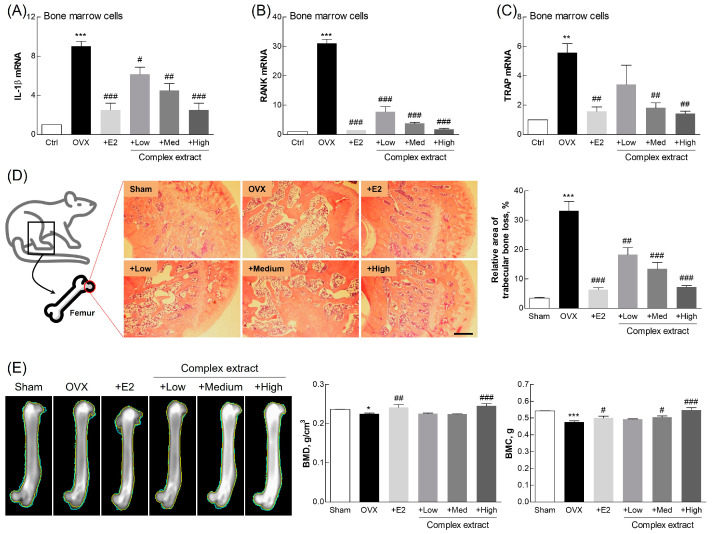
Bone features and gene expression profiles in ovariectomized (OVX) rats treated with the *Styphnolobium japonicum* fruit (SJF) and germinated soybean embryo (GSE) complex extract. (**A**–**C**) Expression of bone resorption-related genes, evaluated using qPCR, in femoral bone-derived bone marrow cells. (**D**) Hematoxylin and eosin-stained transverse sections of femur (left panel). Trabecular bone loss area (right panel). (**E**) Representative X-ray images of femoral bone (left panel). Bone mineral density (BMD) and bone mineral content (BMC) (right panel). Scale bar, 500 µm. The complex extract was prepared by blending the SJF and GSE. 17β-estradiol (E2) was used as a positive control. The results are presented as means ± standard deviation (*n* = 3). * *p* < 0.05, ** *p* < 0.01, *** *p* < 0.001, vs. the sham group; # *p* < 0.05, ## *p* < 0.01, ### *p* < 0.001, vs. the OVX group. IL-1β, interleukin 1β; RANK, receptor activator of nuclear factor κB; TRAP, tartrate-resistant acid phosphatase.

We investigated the effects of the SJF–GSE extract on bone features in the OVX rats, focusing on (1) the expression of genes related to bone resorption and formation, (2) histological changes, and (3) and bone mineralization (Figure 8). The decline in estrogen levels following menopause boosts the formation of osteoclasts, bone-resorbing cells involved in bone remodeling [46]. We observed significant downregulation in the expression of RANK and interleukin-1β (IL-1β), markers associated with osteoclast activity [47], in all of the extract-treated groups, suggesting a reduction in bone resorption (Figure 8A,B). The expression of tartrate-resistant acid phosphatase (TRAP), which is indicative of osteoclast differentiation [48,49], was downregulated in the extract-treated groups, indicating the potential of the complex extract to promote bone health by reducing osteoclastogenesis (Figure 8C). Histological assessment via H&E staining revealed significant preservation of bone density by the extract complex, indicating its efficacy in mitigating osteoporotic changes and estrogen deficits (Figure 8D).

BMC and BMD were examined using X-ray imaging to visualize changes in bone structure. This revealed substantially greater bone density at higher extract concentrations (Figure 8E). The complex extract-treated group (and especially the High-dose group) exhibited higher BMC and BMD than the OVX group, indicating improved bone strength and reduced fracture risk. These findings, consistent with prior findings, support the osteoprotective effects of phytoestrogen [25]. This further confirms the phytoestrogen-like action of the complex extract [50,51].

These findings reveal the benefits and potential therapeutic value of the SJF–GSE complex extract on bone health and in the alleviation of estrogen depletion-associated postmenopausal symptoms. This product, which substantially downregulated osteoclastogenesis-related gene expression and improved the histological structure and mineralization profiles represents an important advancement in developing efficacious treatments for postmenopausal conditions.

Notably, it is important to acknowledge that the biological effects observed in our study are likely influenced by the inherent complexity of the extracts. These extracts are not simple one-component systems but rather complex mixtures of various biomolecules, each contributing to the overall activity. Interactions among these components can lead to synergistic or additive effects, enhancing the therapeutic potential of the extracts [52]. Conversely, antagonistic interactions may also occur, where certain components could suppress the activity of others, impacting the overall efficacy [53]. Moreover, the biological effects of these extracts are closely tied to the extraction methods used, as these methods determine the final composition and activity of the extracts. Although our study primarily focused on the overall effects of the extracts, future research should aim to dissect these interactions more thoroughly to better understand how they contribute to the observed outcomes.

## 4. Conclusions

The complex extract of *S. japonicum* and germinated soybean embryo demonstrated significant therapeutic efficacy in alleviating postmenopausal symptoms, including improved bone density, reduced vasomotor symptoms, and enhanced vaginal health. The extract’s estrogen-like effects and antioxidant properties highlight its potential as a natural alternative to hormone replacement therapy. These findings support the development of plant-based therapies for managing menopausal symptoms.

## Figures and Tables

**Table 1:** Antioxidant, anti-inflammatory, and osteoprotective activity of *Styphnolobium japonicum* fruit extract (SJFE), germinated soybean embryo extract (GSEE), and their combinations.

Activity	SJFE	GSEE	SJFE and GSEE Combinations		
			1:1	1:2	2:1
DPPH scavenging (IC_50_, µg)	61.42 ± 11.23	293.75 ± 15.71	117.56 ± 12.04	84.48 ± 13.74	77.72 ± 14.13
NO inhibition (ED_50_, µg/mL)	206.71 ± 18.11	34.22 ± 5.71	64.82 ± 1.95	48.19 ± 1.88	59.37 ± 1.44
RANKL inhibition (*p* < 0.001, µg/mL)	250	10	100	10	10

The values represent the means ± standard deviation. These results highlight the efficacy of the extracts in scavenging DPPH radicals, inhibiting NO production, and inhibiting RANKL—key indicators of antioxidant, anti-inflammatory, and osteoprotective effects, respectively. For further details, see Figures S2–S4. DPPH, 2,2-diphenyl-1-picrylhydrazyl; NO, nitric oxide; RANKL, receptor activator of nuclear factor κB (RANK) ligand.

**Table 2:** Blood biochemical indices in ovariectomized (OVX) rats treated with the *Styphnolobium japonicum* fruit (SJF) and germinated soybean embryo (GSE) complex extract.

Parameter	Sham	OVX	+E2	Complex Extract		
				+Low	+Medium	+High
E2 (pg/mL)	24.68 ± 1.14	14.4 ± 0.63 *	29.72 ± 1.4 ^##^	19.86 ± 1.5	22.47 ± 2.29	33.59 ± 1.88 ^##^
CTx (pg/mL)	292.97 ± 33.6	548.31 ± 28.81 **	293.39 ± 21.57 ^##^	365.98 ± 49.36 ^#^	264.35 ± 12.44 ^##^	196.32 ± 19.91 ^##^
OC (pg/mL)	7.44 ± 0.34	13.31 ± 0.51 **	5.11 ± 0.7 ^###^	9.44 ± 0.57 ^#^	7.57 ± 0.58 ^##^	6.29 ± 0.17 ^###^
Ca (mg/dL)	10.45 ± 0.05	10.55 ± 0.55	11.15 ± 1.25	10.2 ± 0.1	10.45 ± 0.05	10.8 ± 0.5
P (mg/dL)	3.65 ± 0.05	2.15 ± 0.15 **	3.4 ± 0.2 ^#^	2.3 ± 0.2	3.35 ± 0.15 ^#^	3.75 ± 0.25 ^##^
TG (mg/dL)	49 ± 5	139.25 ± 0.75 **	82.9 ± 7.1 ^#^	94.4 ± 13.6	74.05 ± 13.45	56.1 ± 2.1 ^#^
HDL (mg/dL)	48.3 ± 1.8	41.75 ± 5.25	40.03 ± 1.02	36.5 ± 1.5	43.5 ± 1.5	50.4 ± 2.6
LDL (mg/dL)	5.5 ± 0.5	10.5 ± 0.5 **	5.5 ± 0.5 ^##^	10.5 ± 0.5	10 ± 1	7.5 ± 0.5 ^#^
ALT (U/L)	70.3 ± 2.8	102.05 ± 2.15 *	88.55 ± 6.15	105.95 ± 4.05	87.95 ± 9.25	63.35 ± 2.05 ^#^
LDH (U/L)	318.25 ± 6.25	945.25 ± 80.25 **	684.5 ± 79.5	396.25 ± 20.25 ^##^	210.5 ± 9.5 ^###^	227.5 ± 57.5 ^###^

OVX rats were orally administered the complex extract at 25 mg/kg (low), 50 mg/kg (medium), and 100 mg/kg for 12 weeks. 17β-estradiol (E2) was used as a positive control. * *p* < 0.05, ** *p* < 0.01, vs. the sham group; ^#^ *p* < 0.05, ^##^ *p* < 0.01, ^###^ *p* < 0.001, vs. the OVX group. E2, estradiol; CTx, C-terminal telopeptide; OC, osteocalcin; Ca, calcium; P, phosphorus; TG, triglycerides; HDL, high-density lipoprotein; LDL, low-density lipoprotein; ALT, alanine aminotransferase; LDH, lactate dehydrogenase.

## Data Availability

The original contributions presented in the study are included in the article/Supplementary Material, further inquiries can be directed to the corresponding author.

## Supplementary Materials

The following supporting items can be downloaded at: https://www.mdpi.com/article/10.3390/nu16193297/s1, Figure S1: Effects of SJFE, GSEE, and their combinations on cell viability; Figure S2: Anti-oxidative effects of SJFE, GSEE, and their combinations, evaluated based on their DPPH radical scavenging activity; Figure S3: Effects of SJFE, GSEE, and their combinations on nitric oxide (NO) production in RAW 264.7 cells; Figure S4: Effect of SJFE, GSEE, and their combinations on RANKL gene expression in MG63 cells; Figure S5: HPLC analysis of the sophoricoside and soyasaponin 1 standards; Figure S6: Organ safety assessment in ovariectomized (OVX) rats treated with the complex extract; Table S1: Primer sequences for gene expression analysis; Table S2: Antibody list for western blot analysis; Table S3: Quantitative analysis of the active compounds in the SJFE and GSEE mixtures and in the final complex extract.
