# Supplementary material for: Styphnolobium japonicum Fruit and Germinated Soybean Embryo Complex Extract for Postmenopausal-Symptom Relief
Source: Nutrients. 2024 Sep 28;16(19):3297. doi: 10.3390/nu16193297 (PMC11478548; doi:10.3390/nu16193297)

Styphnolobium japonicum fruit and germinated soybean embryo complex extract for postmenopausal-symptom relief

Jeong Won Ahn[1†], Hyun Soo Kim[1†], Kongara Damodar[2,3], Hee Hyun Shin[4], Kyung Mi Kim[4], Jung Youl Park[5], Su Kil Jang[1,3], Yeong Min Yoo[2], Jae Chul Jung[4], and Seong Soo Joo[1,3*]

[1]Department of Marine Bioscience, College of Life Science, Gangneung-Wonju National University, Gangneung 25457, Gangwon, Republic of Korea

[2]East Coast Life Sciences Institute, College of Life Science, Gangneung-Wonju National University, Gangneung 25457, Gangwon, Republic of Korea

[3]Huscion MAJIC R&D Center, 331 Pangyo-ro, Seongnam 13488, Gyeonggi, Republic of Korea

[4]Life Science Research Institute, NOVAREX Co., Ltd., Cheongju 28220, Chungbuk, Republic of Korea

[5]Glocal University Project Group, Andong National University, 1375 Gyeongdong-ro, Andong, Gyeongsangbuk-do 36729, Republic of Korea

[†] Equally contributed to this study

* Correspondence: ssj66@gwnu.ac.kr (S.S.J.); Tel.: +82-33-640-2856; Fax: +82-33-640-2849

Authors: Jeong Won Ahn, 0000@gwnu.ac.kr; Hyun Soo Kim, gustn1321@nate.com; Kongara Damodar, kongaradamu@gwnu.ac.kr; Hee Hyun Shin, hhshin@novarex.co.kr; Kyung Mi Kim, kkm3507@novarex.co.kr; Jung Youl Park, jypark09@anu.ac.kr; Su Kil Jang, waterroad79@hanmail.net; Yeong Min Yoo, yyeongm@hanmail.net; Jae Chul Jung, jcjung@novarex.co.kr.

Table S1. Primer sequences for gene expression analysis. Primers were selected for genes involved in bone metabolism and inflammatory response in rats, and estrogen response in humans. RANKL, receptor activator of nuclear factor κB (RANK) ligand; TRAP, tartrate-resistant acid phosphatase; IL, interleukin.

Gene		Primer	Sequences	Accession No.
Human	β-actin	5’ primer	5´-AGAGCTACGAGCTGCCTGAC	NM_001101.5
		3’ primer	5´-AGCACTGTGTTGGCGTACAG	
	pS2	5’ primer	5´-CCATGGAGAACAAGGTGATC	NM_003225.3
		3’ primer	5´-TTAGGATAGAAGCACCAGGG	
	RANKL	5’ primer	5´-TGAGACTCCATGAAAATGCA	NM_003701.4
		3’ primer	5´-CGGTGGCATTAATAGTGAGA	
Rat	β-actin	5’ primer	5’-CATCAAAGAGAAGCTGTGCT	NM_031144.3
		3’ primer	5’-GAAGGAAGGCTGGAAAAGAG	
	TRAP	5’ primer	5’-GAAACCATGATCACCTTGGC	XM_003752236.2
		3’ primer	5’-CAGCATAAAGATGGCCACAG	
	RANK	5’ primer	5’-GCGTTTACTACAGGAAGGGA	XM_008769487.2
		3’ primer	5’-CCTTCACACACTTCTTGCTG	
	IL-1β	5’ primer	5’-ATGAGGACCCAAGCACCTTC	NM_031512.2
		3’ primer	5’-CAGACAGCACGAGGCATTTT	

Table S2. Antibody list for western blot analysis. Antibodies were selected for proteins involved in estrogen receptor-mediated signaling pathways. ER, estrogen receptor; HRP, horseradish peroxidase.

<table>
<tr><th colspan="2">Epitope</th><th>Manufacturer</th><th>Cat. No.</th><th>Dilution</th><th>Host</th></tr>
<tr><td rowspan="4">Primary</td><td>ERα
(70 kDa)</td><td>FineTest</td><td>FNab02822</td><td>1:1000</td><td>Rabbit</td></tr>
<tr><td>Akt1
(60 kDa)</td><td rowspan="3">Santa Cruz Biotechnology</td><td>sc-8312</td><td>1:1000</td><td>Rabbit</td></tr>
<tr><td>p-Akt1
(60 kDa)</td><td>sc-101629</td><td>1:1000</td><td>Rabbit</td></tr>
<tr><td>β-actin
(43 kDa)</td><td>sc-81178</td><td>1:1000</td><td>Mouse</td></tr>
<tr><td rowspan="2">Secondary
(HRP-linked)</td><td>Mouse IgG</td><td rowspan="2">Cell Signaling Technology</td><td>#7076</td><td>1:10000</td><td>Goat</td></tr>
<tr><td>Rabbit IgG</td><td>#7074</td><td>1:10000</td><td>Horse</td></tr>
</table>

Table S3. Quantitative analysis of the active compounds in the SJFE and GSEE mixtures and in the final complex extract. High-performance liquid chromatography was used to analyze sophoricoside and soyasaponin 1 in the test substances. The complex extract was prepared by blending SJE and GSE at a ratio of 1.5:1. Data from three independent experiments are shown as means ± standard deviation. SJFE, *Styphnolobium japonicum* fruit (SJE) extract; GSEE, germinated soybean embryo (GSE) extract.

Extracts		Active compounds (mg/g)	
		Sophoricoside	Soyasaponin 1
SJFE and GSEE mixture (ratio)	1:1	48.64 ± 1.02	7.39 ± 1.10
	1:2	33.52 ± 0.78	5.51 ± 0.87
	2:1	63.06 ± 1.26	9.58 ± 1.55
Complex extract		59.25 ± 0.68	9.09 ± 2.84

Figure S1. Effects of SJFE, GSEE, and their combinations on cell viability. Three distinct cell lines were used in this study. Cell viability was measured using WST-8 assay in RAW 264.7 macrophages (A), MG63 osteoblasts (B), and MCF7 breast cancer cells (C). A 10 mM hydrogen peroxide (H_2O_2) was used as a positive control. Data from three independent experiments are shown as means ± standard deviation. Statistical significance is denoted by * $p < 0.05$, *** $p < 0.001$, compared to the Ctrl group. SJFE, *Styphnolobium japonicum* fruit extract; GSEE, germinated soybean embryo extract.

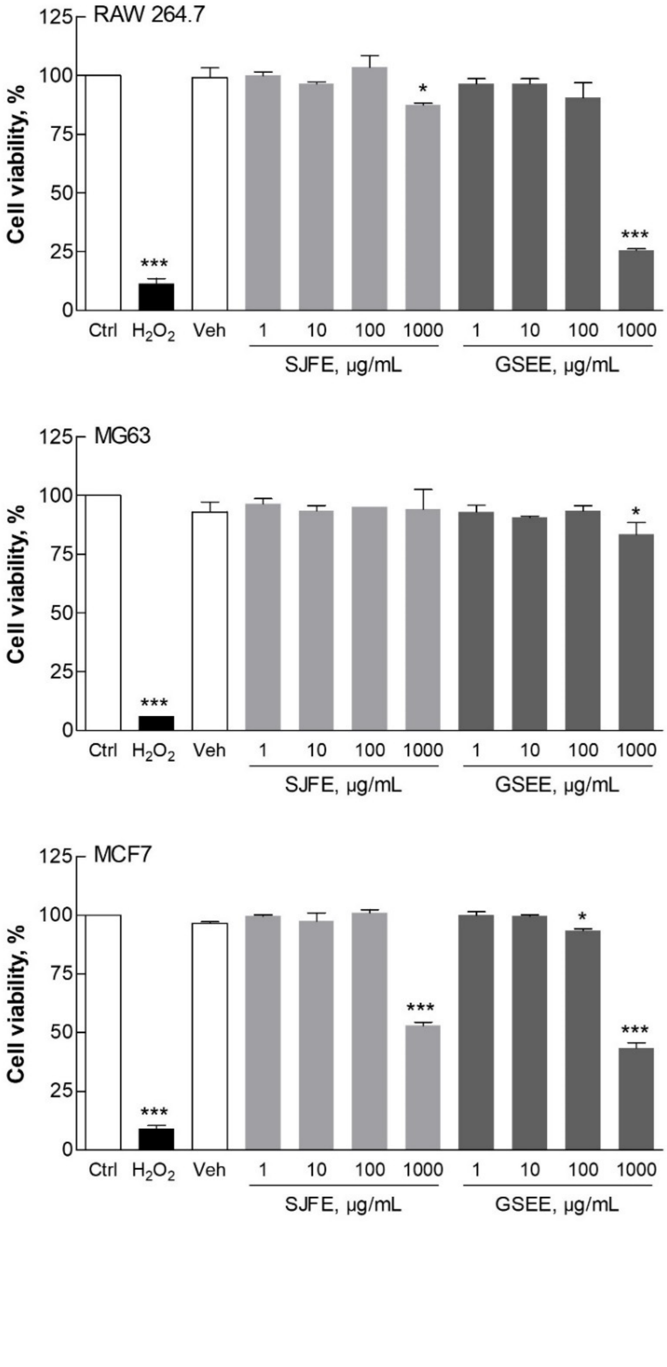

Figure S2. Anti-oxidative effects of SJFE, GSEE, and their combinations, evaluated based on their DPPH radical scavenging activity. The test substances were incubated with a 0.2 mM DPPH solution at different capacities ranging from 10 to 100 μg. The degree of radical scavenging was determined by measuring the change in absorbance at 517 nm. A 100 μM ascorbic acid (Asc) was used as a positive control. Data from three independent experiments are shown as means ± standard deviation. Statistical significance is denoted by * $p < 0.05$, ** $p < 0.01$, *** $p < 0.001$ compared with the Ctrl group. SJFE, *Styphnolobium japonicum* fruit extract; GSEE, germinated soybean embryo extract; DPPH, 2,2-diphenyl-1-picrylhydrazyl.

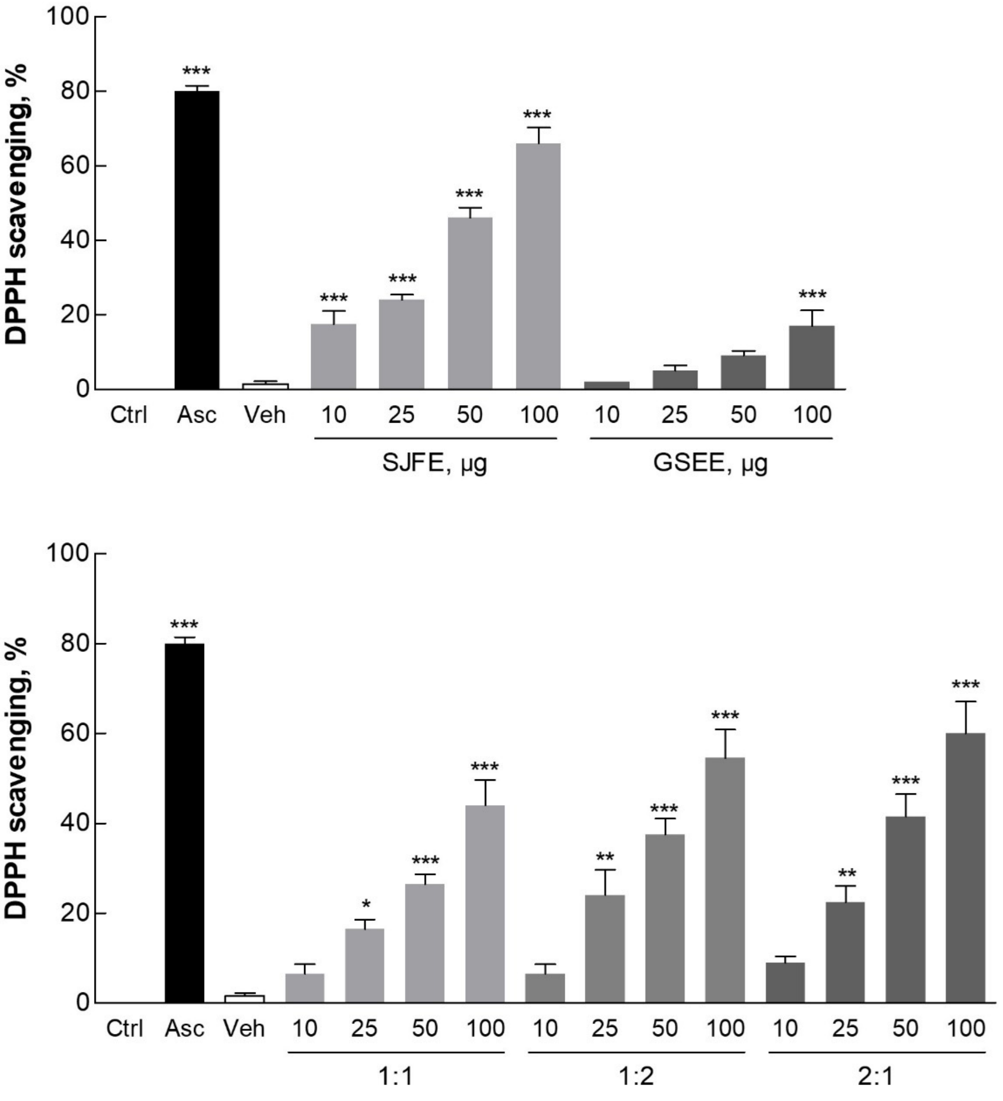

Figure S3. Effects of SJFE, GSEE, and their combinations on nitric oxide (NO) production in RAW 264.7 cells. The cells underwent treatment with concentrations ranging from 10 to 100 μg/mL of the test substances for 1 h, followed by a 20-hour stimulation with 1 μg/mL of LPS. NO production levels were quantified using the Griess reagent assay. Data from three independent experiments are shown as means ± standard deviation. Statistical significance is denoted by *** $p < 0.001$ compared with the Ctrl group; # $p < 0.05$, ## $p < 0.01$, ### $p < 0.001$ compared with the LPS treated group. SJFE, *Styphnolobium japonicum* fruit extract; GSEE, germinated soybean embryo extract; LPS, lipopolysaccharide.

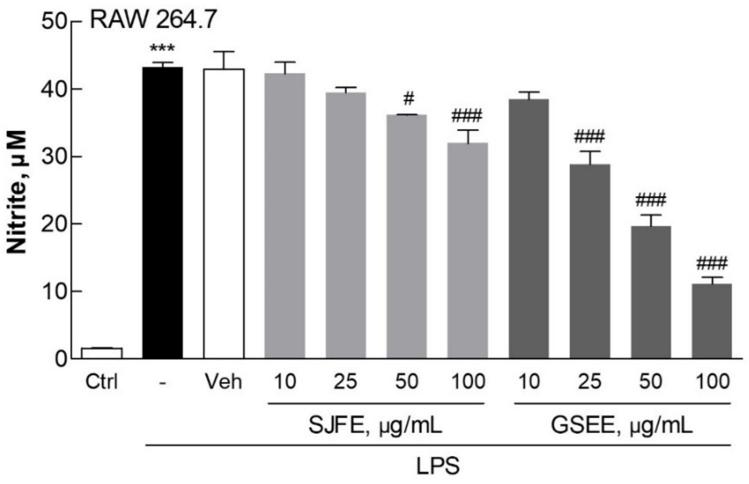

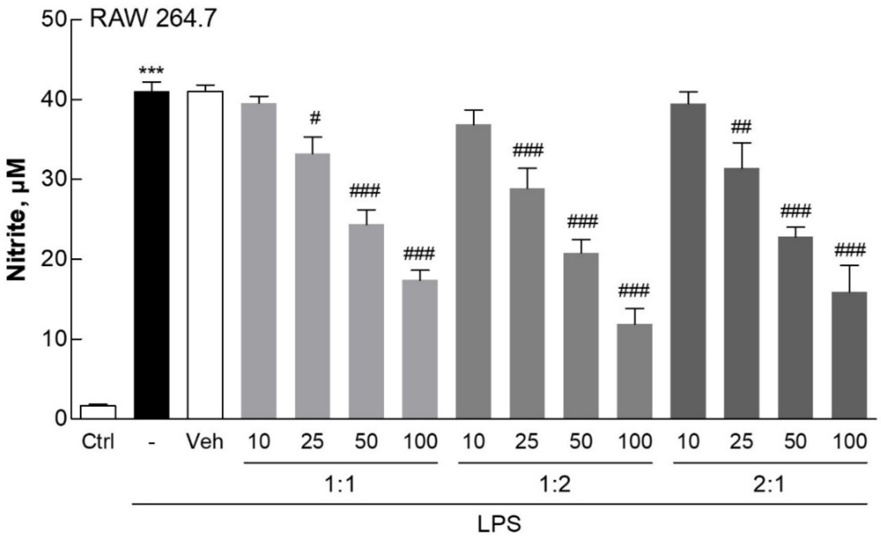

Figure S4. Effect of SJFE, GSEE, and their combinations on RANKL gene expression in MG63 cells. The cells were initially treated with varying concentrations of the test substances for 1 h, then exposed to LPS-stimulated RAW 264.7 cell media (RM) for 20 h. RANKL gene expression levels were quantitatively assessed using quantitative polymerase chain reaction. Data from three independent experiments are shown as means ± standard deviation. Statistical significance is denoted by *** $p < 0.001$ compared with the Ctrl group; ## $p < 0.01$, ### $p < 0.001$ compared with the RM treated group. SJFE, *Styphnolobium japonicum* fruit extract; GSEE, germinated soybean embryo extract; LPS, lipopolysaccharide; RANKL, receptor activator of nuclear factor κB ligand.

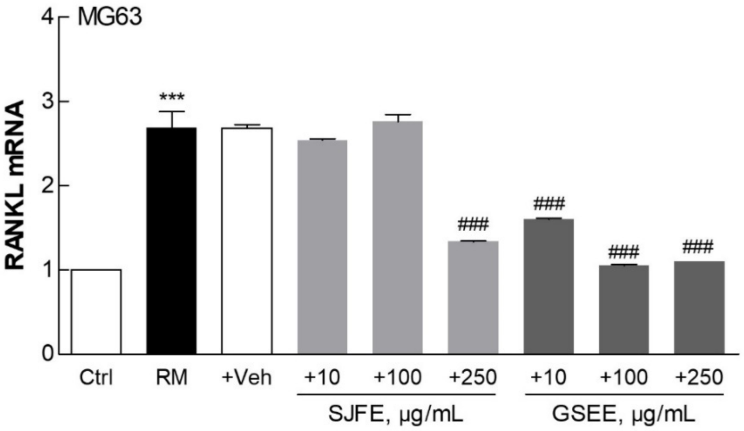

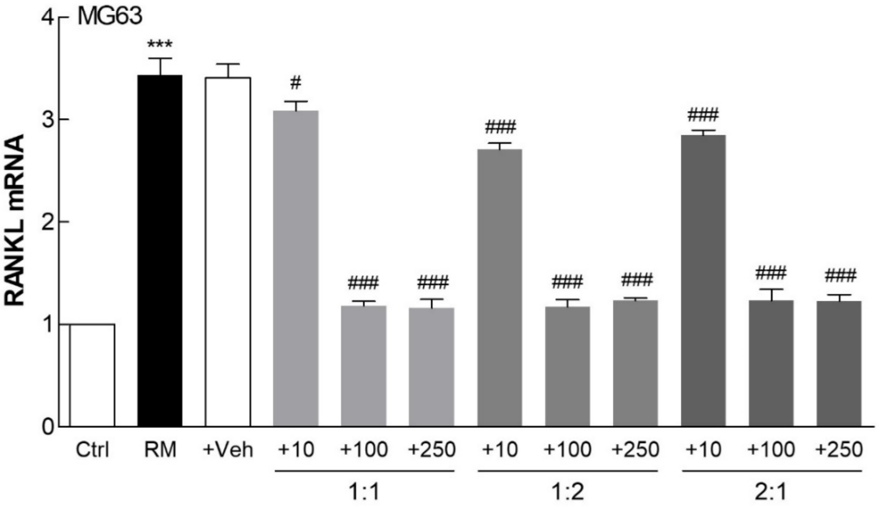

Figure S5. HPLC analysis of the sophoricoside and soyasaponin 1 standards. The figure displays a representative HPLC chromatogram (*left panel*) and calibration curves (*right panel*) for each compound: sophoricoside (A) and soyasaponin 1 (B). Sophoricoside was detected at a retention time (RT) of 15.0 min, while soyasaponin 1 was detected at a RT of 17.2 min.

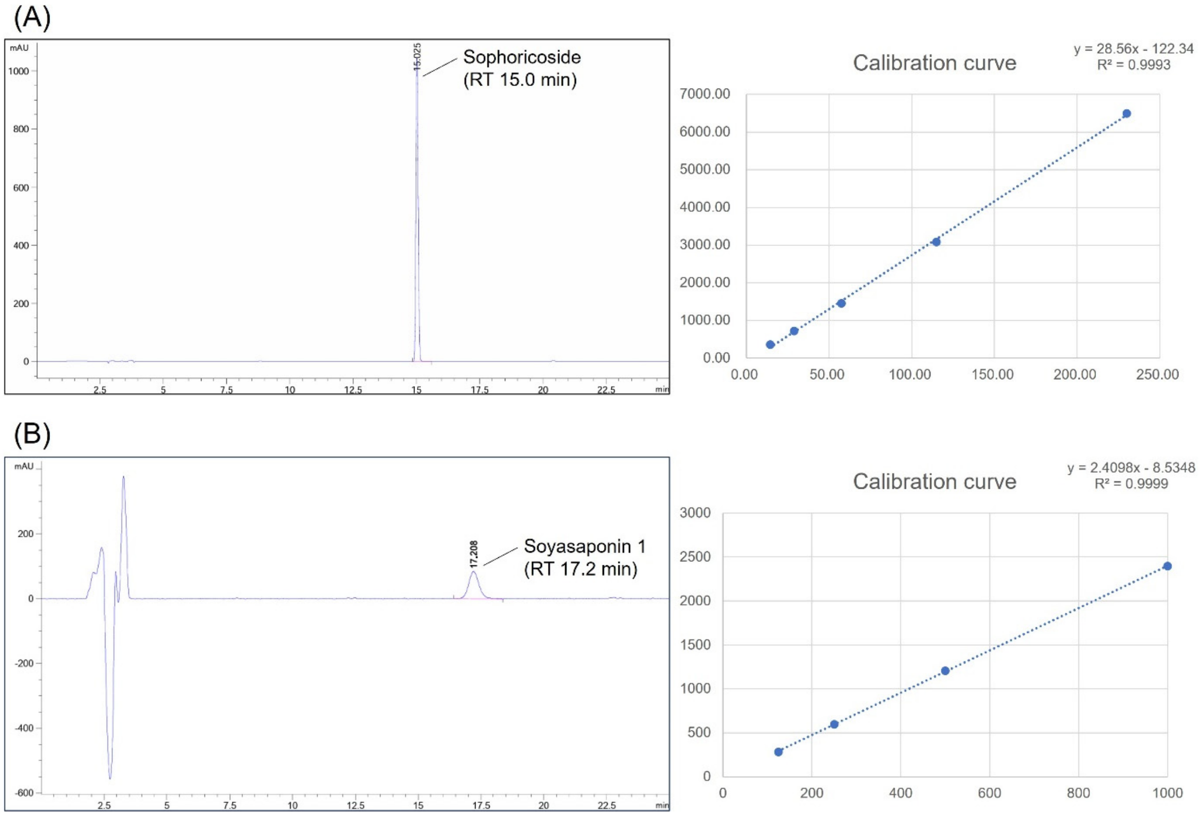

Figure S6. Organ safety assessment in ovariectomized (OVX) rats treated with the complex extract. (A) Representative images of major organs, including the liver, spleen, kidney, and stomach, excised after 12 weeks of treatment. (B) Depicts a comparison of the tissue mass for each organ. The complex extract was prepared by blending SJF and GSE at a ratio of 1.5:1. 17β-estradiol (E2) was used as a positive control. The scale bar in the images represents 1 cm. SJF, *Styphnolobium japonicum* fruit; GSE, germinated soybean embryo.

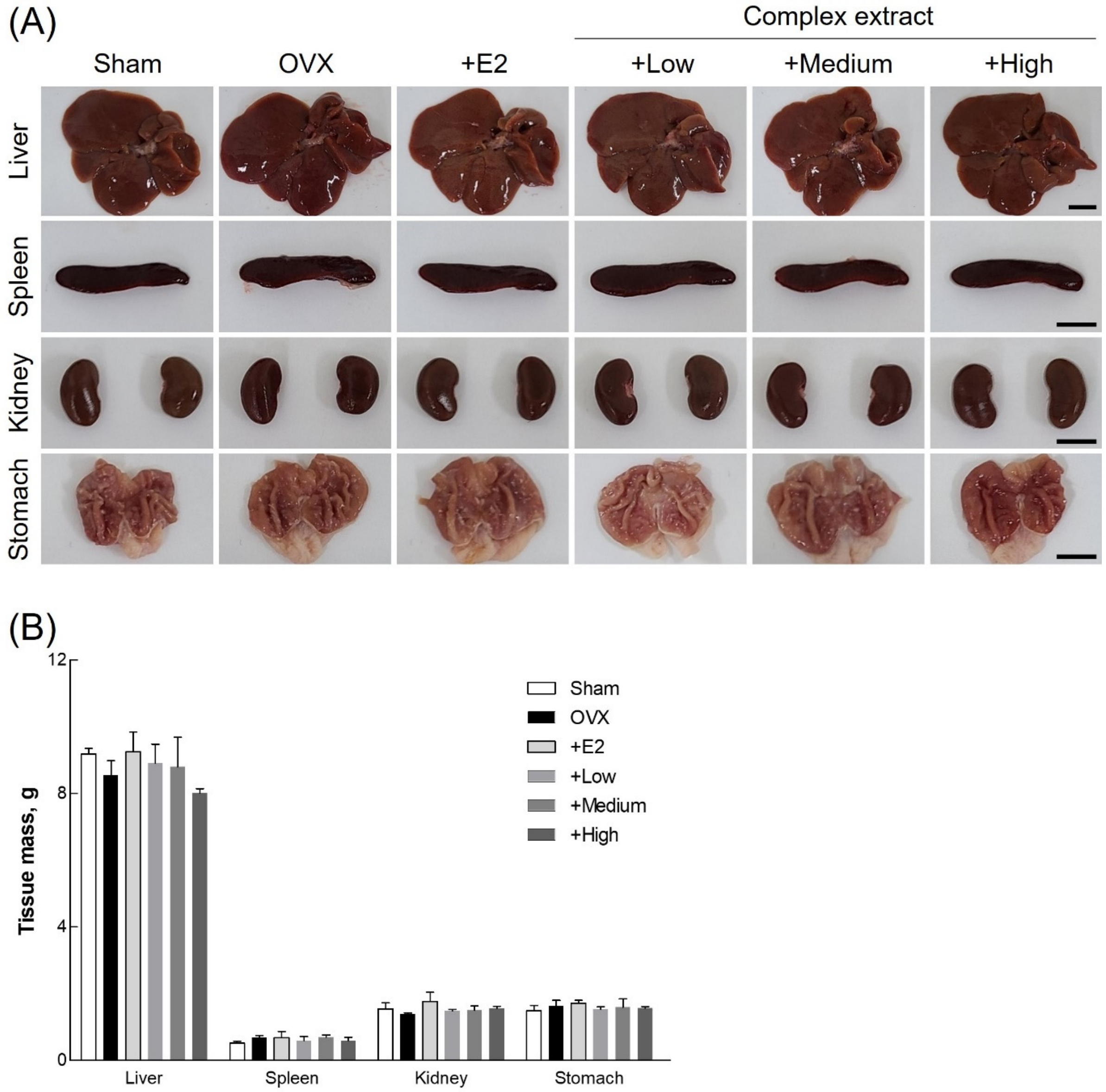

Supplement: Supplementary file 1 [file nutrients-16-03297-s001.zip › nutrients-3225719-supplementary.pdf]
